# Lung resection in pulmonary aspergilloma: experience of a Moroccan center

**DOI:** 10.1186/s12893-015-0103-4

**Published:** 2015-10-16

**Authors:** Mohammed Massine El Hammoumi, Omar Slaoui, Fayçal El Oueriachi, El Hassane Kabiri

**Affiliations:** Department of Thoracic Surgery, Mohamed V Military University Hospital, Riad 10100, Rabat, Morocco; Center of doctoral studies, Faculty of Medecine and Pharmacy, Mohamed V University, Rabat, Morocco

**Keywords:** Aspergilloma, Surgery, Hemoptysis, Lobectomy, Lung infection

## Abstract

**Background:**

This study was conducted to determine the efficacy of surgery in the treatment of complex aspergilloma comparatively with simple aspergilloma.

**Methods:**

From January 2006 to December 2014, 115 cases of pulmonary aspergilloma were admitted in our department. One operation on one side was counted as one case and the patients were divided into two groups. In group A: 61 cases of complex aspergilloma. In group B: 50 patients underwent 54 cases of lung resection for simple aspergilloma. People who underwent arteriography and embolization were excluded. Surgical treatment was indicated when 1) recurrent aspergilloma-related hemoptysis, 2) definite simple or complex aspergilloma and 3) a simultaneous bilateral aspergilloma.

**Results:**

People with complex aspergilloma were big smokers with lower BMI, and had reduced lung function parameters. The main symptoms were repeated hemoptysis, chronic cough, abundant purulent expectoration and respiratory infections. Lobectomy was the most performed indication. In group B, number of wedge resections was larger than group A with statistical significant difference (*p* = 0.001). In the post-operative course morbidity was higher in group A (16 %) vs (9 %) in group B with statistical difference (*p* = 0.026). The median follow-up was 30 months (range 19–52 months).

The median duration of chest tube drainage was 4 days. The duration of chest tube drainage was longer in the group A (4.7 ± 1.4 versus 2.9 ± 1.3; *p* = 0.005). The prolonged postoperative air leakage occurred more frequently in group A (14.75 %; versus 1.8 % *p* = 0.015). In group A, 3 cases and 2 in group B underwent a secondary operation for post operative hemothorax. Bronchopleural fistula occurred exclusively in group A (*n* = 4).

**Conclusions:**

The surgical resection should be used in a multidisciplinary approach. Preoperative Interventional therapies could optimize the conditions for the operation. Total surgical resection must be the treatment of choice of localized causative lesions.

## Background

Aspergillus fumigatus is a common saprophytic fungi causative of a large spectrum of diseases. Pulmonary aspergillosis range from a benign aspergillosis: aspergilloma, to invasive aspergillosis, which is a fatal form and usual complication in immunocompromised patients. Aspergilloma results from local colonization of 10 to 20 % of lung tuberculosis cavities. Less frequently, cavities resulting from emphysema bubbles, necrotizing infection, bronchiectasis, sarcoidosis, radiotherapy and neoplastic cavitation [1, 2].

Pulmonary aspergilloma can be classified into: simple form presenting as an isolated cavity with thin walls, surrounded by normal lung parenchyma and more frequently complex form, where cavities have thick walls surrounded by fibrotic lung tissue, stiff hilar structures, vascular adhesions and obliteration of the pleural cavity. Lung resection of a symptomatic intracavitary aspergilloma is a curative approach but not always feasible in those with compromised pulmonary function and or extensive bilateral pulmonary disease. Therefore some surgical procedures such as cavernostomy are necessary [3]. In this study, we retrospectively investigated the effectiveness of lung resection and analyzed our results.

## Methods

**Fig. 1:**
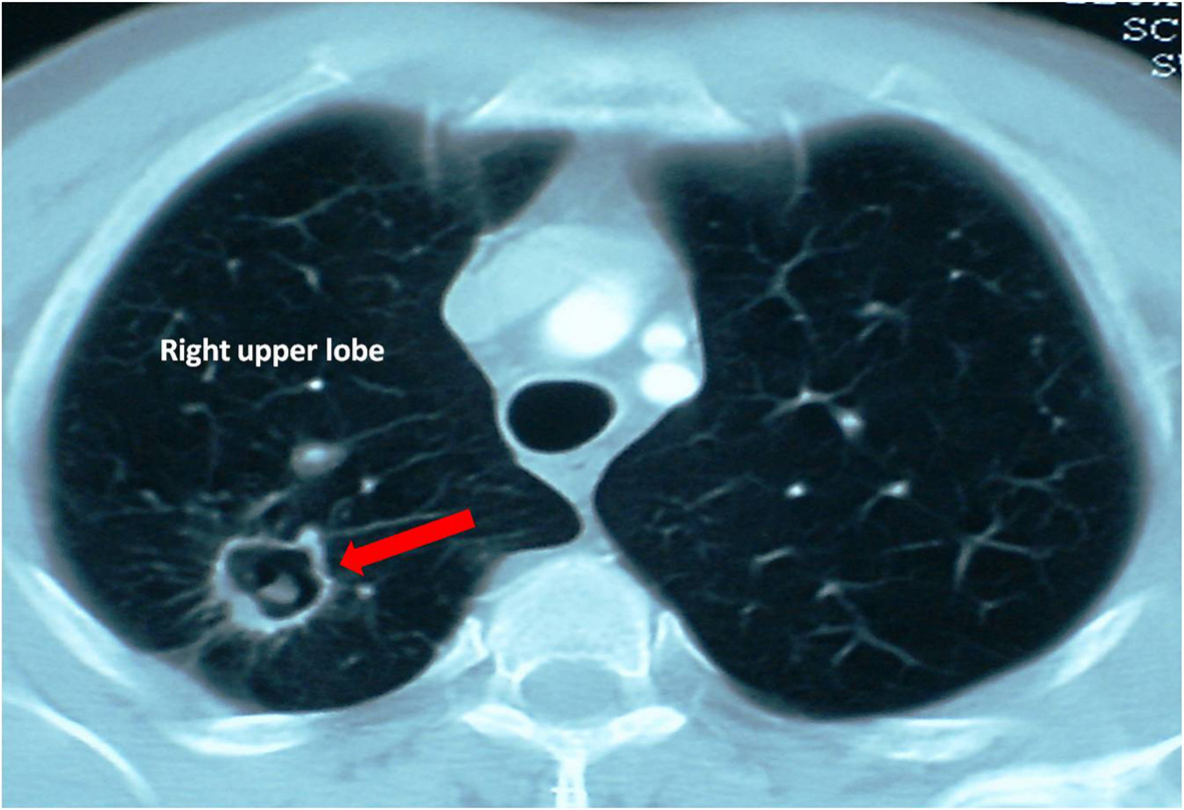
Computed tomographic view of a simple aspergilloma of the right lung

**Fig. 2:**
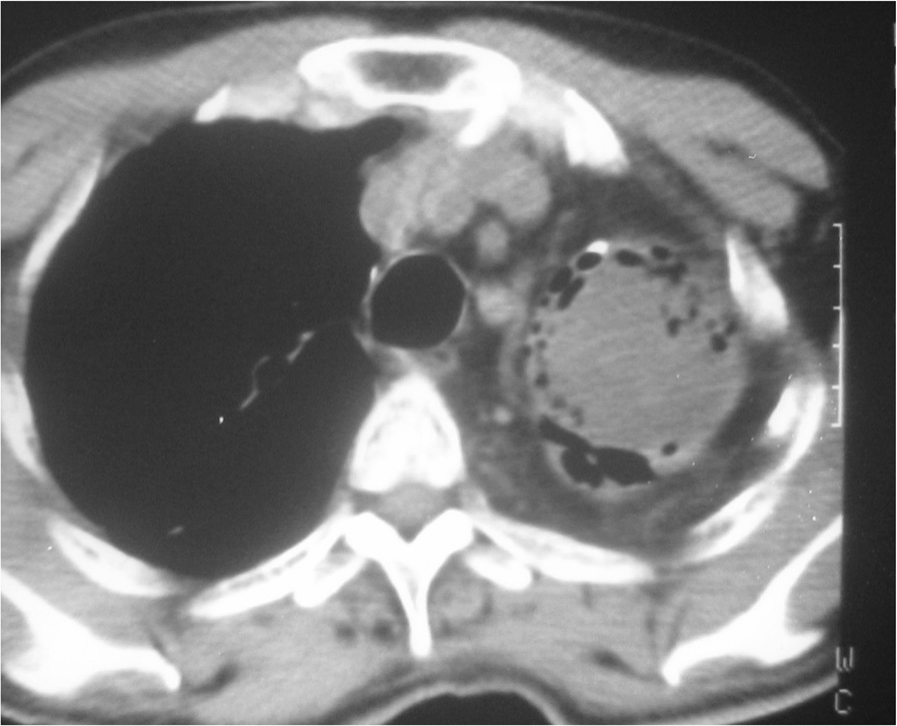
Computed tomographic view of a complex aspergilloma of the left lung

From January 2006 to December 2014, 274 cases of pulmonary aspergilloma were admitted in our hospital. One hundred Fifty nine cases from the initial study population were excluded. The following criteria were used to exclude patients from this study: 1) patients who had poor general state, 2) patients who had a history of advanced associated cancer, 3) patients who had limited respiratory function, and 4) patients who had extensive bilateral disease (Table 1). One operation on one side was counted as one case and the patients were divided into two groups based on initial diagnosis and according to the classification of Plummer and Belcher of 1960 [2]. In group A: 61 cases of complex aspergilloma (Fig. 1). In group B: 50 patients underwent 54 cases of lung resection for simple aspergilloma (Fig. 2). Patient characteristics are presented in Table 2. People who underwent arteriography and embolization were also excluded because this technique is performed in another department and we couldn’t control the chart review.

**Table 1:** Flow diagram of population study

Eligible Patients with lung aspergillosis lesions *N* = 274	
Included: *n*= 115	Excluded: *n*= 159
-Patient with asergilloma lesions and colmplete CT follow -up	-Patients with poor general state (N=25)
	-Patient with limited respiratory function (*N*= 65)
	-Patient with malignancy: (*N*=6)
	-Absence of follow up (*N*=42)
	-Initial invasive diffuse aspergillosis (N=21)

**Table 2:** Baseline characteristics of study subjects

Variables	Group A (*n*=61)	Group B (*n*=50)	*P* value
Sexe			
male	51	42	0.502
female	10	08	
Age (years)	39.5±5.2	37.8±3.8	0.225
BMI* (KG/m^2^)	18.8± 2.9	22.8±2.6	0.002
History of smoking			
never	19	24	0.425
Ex	16	11	0.269
Unknown	26	15	0.013
Pack-years	18	10	0.002
Comorbidities: tuberculosis COPD, diabete	18	06	0.025
	12	03	0.004
Pulmonary function test (FEV* L, % predicted)	1.8±0.56	2.0±0.79	0.0015
	68 % ±15 %	80 % ±10 %	
Aspergilloma Side			
Right	39	29	0.356
left	22	19	0.247
bilateral	0	2	0.350

We declare that this study is approved by the ethics committee of Mohammed V Military teaching Hospital, and total written consent was obtained from all participants.

### Surgical indications

Surgical treatment of pulmonary aspergilloma was offered when patients had: 1) recurrent aspergilloma-related hemoptysis, 2) definite simple or complex aspergilloma that were detected on chest X-ray or on a computed tomography scan, and 3) a simultaneous bilateral aspergilloma.

To prevent life threatening complications in asymptomatic patients, we are convinced that a scheduled surgery should be performed.

### Operation procedures

All patients received general anesthesia and were intubated with a double-lumen endotracheal tube to allow selective lung ventilation. The patients were placed in the lateral decubitus position. Operation procedures were as follows: Thoracoscopic approach with wedge resection for simple peripheral lesions and classical thoracotomy with segmentectomy, lobectomy or pneumonectomy for complex lesion. Cavernostomy was performed when the respiratory function was limited. Myoplasty was performed in the same time when a post-operative residual cavity was predictable. After coagulation of the bleeding two 28 Fr and 32 Fr chest tubes were inserted. After pneumonectomy we used to insert one chest tube for 24 h without sucction.

### Postoperative chest tube management

Except for pneumonectomy, negative suction was applied immediately after operation and maintained until chest tube removal. The chest tube was removed after: cessation of air leakage with drainage of less than 100 ml for 24 h, or complete resolution of the residual pneumothorax.

### Statistical analysis

All values in the text and tables were expressed as mean ± SD. The data were analyzed using the software Statistical Package for the Social Sciences (IBM SPSS Statistics, version 15, SPSS Inc.) system. Unpaired *t*-test, *χ*^2^ test, and Fisher exact test were used for group differences. A *p* value less than 0.05 was considered statistically significant.

## Results

Demographic characteristics of group A and B are presented in Table 2. There was no difference in sex, age and aspergilloma site. But a significant difference was noted in body mass index, comorbidities, lung function test and pack-years smoking. People with complex aspergilloma were big smokers with lower BMI, and had reduced lung function parameters. The main complaints were: repeated pulmonary bleeding or sputum, hemoptysis with volume ranging from 400 ml and 750 ml in 24 h, chronic cough, abundant purulent expectoration and respiratory infections. Eleven patients had digital clubbing. The main indication and type of surgery were resumed in Tables 3 and 4. In the post-operative course mortality and morbidity are reported in Table 5. The median follow-up was 30 months (range 19–52 months).

**Table 3:** Main indications of surgery in aspergilloma

Surgical indication	Group A	Group B	*P* value
Recurrent hemoptysis	50 (81.96 %)	38 (76 %)	0.413
Emergency surgery	04 (6.55 %)	0	0.145
Destroyed parenchyma	39 (63.93 %)	12 (24 %)	0.032
Recurrent infection	42 (68.85)	29 (58 %)	0.524
Bilateral aspergilloma	0	2 (4 %)	0.350

**Table 4:** Surgical performed procedures in aspergilloma cases

Surgery	Group A (*n*=61)	Group B (*n*=54)	*P* value
Wedge resection	16	36	0.001
Segementectomy by VATS	0	2	0.512
Lobectomy	27	15	0.263
pneumonectomy	11	0	0.026
cavernostomy	2	0	0.362
Myoplasty after lobectomy	5	1	0.003

**Table 5:** Postoperative morbidity and mortality

Post operative complications	Group A(*n*=61) (% of the group)	Group B (*n*=54) (% of the group)	*P* value
Mortality	2 (3.27 %)	0	0.312
pyothorax	4 (6.55 %)	2 (3.7 %)	0.257
Residual cavity	9 (14.75 %)	1 (1.8 %)	0.015
Bronchopleural fistula	4 (6.55 %)	0	0.311
hemothorax	3 (4.9 %)	2 (3.7 %)	0.353
Total morbidity	16 ( 26.6 %)	5 (9.25 %)	0.026

In group B, number of wedge resections was larger than group A with statistical significant difference (*p* = 0.001).

The median duration of chest tube drainage was 4 days. The duration of chest tube drainage was longer in the group A (4.7 ± 1.4 versus 2.9 ± 1.3; *p* = 0.005). The prolonged postoperative air leakage occurred more frequently in group A (14.75 %; versus 1.8 % *p* = 0.015).

In group A, 3 cases underwent a secondary operation for post operative hemothorax. Two of patients in group B required another operation for the same reason (Table 5).

Two of the three re-operated patient in group A died on early postoperative course, owing to respiratory and heart insufficiency after a pneumonectomy.

Bronchopleural fistula occurred in 4 cases 3.4 % (3 after pneumonectomy and one after a lobectomy exclusively in the group A).

### Follow-up

Twenty one (18 %) patients were lost to follow-up, and 94 (81.7 %) patients had regular follow-up. The end-point of follow-up was December 2014. The median period of follow-up was 21.5 months (range, 1–51 months; IQR, 6.75–35.25 months).

No recurrence of aspergillosis in both groups was noted after 1 year of postoperative follow-up.

## Discussion

In 1960, Belcher and Plummer [4], presented a clinical and radiological classification which seems definitive, since it still applies 45 years later! These authors distinguished the simple to the complex aspergilloma.

Radiologically, complex aspergilloma is characterized by parenchymal excavation thick edges, associated with pulmonary fibrosis and a pleural thickening. This form of presentation is typically post tuberculous cavity. In contrast, simple aspergilloma is a parenchymal cavity with thin edges, without associated pleural or parenchymal lesions.

Clinically, Patients with a simple aspergilloma are often asymptomatic and have no functional or nutritional tare. The prevalence of asymptomatic patients can vary from 18 to 22 % [5]. In contrast, patients with complex aspergilloma are in poor condition, and nutritionally deficient. They are usually symptomatic, with hemoptysis and bronchorrhea [6, 7]. Their respiratory function is reduced.

In the current literature there is no consensus about the timing of the surgical resection in cases of lung aspergilloma. But since the hemorrhage comes from the bronchial artery system in majority of patients, embolization may control the hemoptysis in only 75 % of cases, with a recurrence rate up to 75 % [8–10]. We were unable to study the results of this method on our patients since the majority of these cases were treated in the department of pulmonology.

Prophylactic lung resection in asymptomatic patients to prevent massive hemoptysis is frequently indicated [11, 12]. Therefore, spontaneous lysis of aspergilloma may occur in only 5–7 % of cases.

Morbidity and mortality rates are higher in emergency cases of pulmonary resections for hemoptysis [13–15], that is why we always tried to stabilize our patients to perform elective and economical lung resection.

Andrejak et al. have categorized resections into three groups as 1) emergency, 2) after bleeding control and 3) planned (after discharge) and reported a series of 111 lung resections for severe hemoptysis out of 813 cases [15]. Mortality ratios were 35, 4 and 0 % among the groups, respectively. This study indicated the importance of avoiding emergent lung resections as much as possible to minimize the morbidity and mortality and to avoid imprecise evaluation of pulmonary functions and other comorbidities.

The ideal surgical treatment consists of an anatomical resection carrying the mycetoma and underlying cavity. The persistence of parenchymal cavity calls for recurrence mycetoma. For this reason, the basic corresponds to a lobectomy, provided it is feasible anatomically and functionally [6, 7, 16]. Segmentectomy exposed to an increased risk of prolonged air leaks, or even residual cavity that could be secondarily contaminated, besides the transparenchymal dissection has a theoric risk of opening of the cavity and intraoperative swarming.

It was suggested that the approach of partial resection was feasible in patients with focal infections including aspergilloma. Many authors performed wedge resection in some cases of lung aspergilloma [16, 17].

Our strategy for small peripheral pulmonary local lesions was partial resection. It is also an acceptable surgical manipulation choice, but maintaining a high margin/lesion ratio to better guarantee operational safety [18].

The safety and efficacy of VATS for pulmonary aspergilloma needs to be analyzed using a larger studies. Considering our experience, VATS can be safely applied to peripheral simple and complex aspergilloma without infiltration of the hilum. In the case of complex aspergilloma close to the hilum a careful consideration is needed, because dissection is obviously difficult and convert to a thoracotomy should be planned. VATS for simple pulmonary aspergilloma, if applicable is a good indication for VATS.

The pneumonectomy is often easily indicated since lung lesions are usually extensive with a high risk of empyema, with or without a bronchial fistula, and should be avoided [19]. Technically resections are difficult and bleeding in complex aspergilloma, due to intense fibrosis installed around the pleural space, interlobular fissures and pulmonary hilum associated to an intense collateral circulation, enlarged and tortuous bronchial arteries. Therefore lobar or segmental pulmonary resection is usually performed in cases of simple aspergilloma.

Conventional surgical complications including intraoperative and postoperative bleeding, prolonged air leakage, indicating secondary thoracoplasty and respiratory distress, are the prerogative of patients with complex aspergilloma. According to the literature excessive bleeding was more observed with complex aspergilloma [5, 6, 20].

This pulmonary resection approach displays operative mortality from 0 to 44 %, and morbidity from 15 to 78 % in accordance to our current results. In our series, number of wedge resections was larger in the group of simple aspergilloma than complex aspergilloma with statistical significant difference (*p* = 0.001) we can then conclude that early diagnosis allow a lung parenchyma sparing. Current studies shows a rarity of complex apergilloma and a predominance of the simple form; the reduction of post-tuberculous sequelae is certainly a major factor [20].

In the literature, the simple aspergilloma operative mortality is similar to the one of the general population [21–25] probably related to the criteria in the selection of patients, adequate lung capacity, the limited underlying lung disease and the extent of pulmonary resection. In our series patients with complex aspergilloma were potentially exposed to post operative complications (16 % vs 9 %) since the BMI, lung function parameters were statistically reduced, comorbidity and smoking related risk were higher than group with simple aspergilloma.

Due to the low number of pneumonectomies in both groups we couldn’t compare pneumonectomy related morbidity and the one of lesser resections.

In our series, people with complex aspergilloma seemed to have more incidence of residual cavity after surgery, this can be easily explained by the poor quality of remaining functional lung and the two died patients were in the postoperative course of a pneumonectomy (j + 1 and J + 3, cardiac failure) after an emergent surgery due to life threatening hemoptysis. This is why the surgical decision of pleuro-pneumonectomy should be well evaluated preoperatively taking in mind that cavernostomy is still a surgical option and a viable alternative, even to those that can be submitted to pulmonary resection, especially in patients with pneumonectomy indication and also in those with bilateral aspergilloma. The cavernostomy is necessary in patients with compromised lung function, with lower morbidity, mortality and functional deficit [11, 23, 24, 26, 27].

Operative techniques of cavernostomy differ after the opening of the cavity and the removal of fungal material [24-26, 28-30]

According to Regnard et al. [19] this procedure was effective in controlling hemoptysis, compared to patients undergoing lobectomy or segmentectomy and the long-term survival was similar. they emphasized that cavernostomy should be remembered, especially when there is indication for pleuropneumonectomy.

## Conclusion

Symptoms were more frequently associated with complex aspergilloma as compared to simple aspergilloma. Surgery for complex aspergilloma was associated with low mortality but significant morbidity, whereas simple aspergilloma had low postoperative morbidity and no mortality.

The surgical resection should be used in a multidisciplinary approach.

Bronchial blockers or arterial embolization can be used preoperatively to optimize the conditions of the operation.

Total surgical resection must be the treatment of choice for all patients with localized lesions.
